# Cross-Cultural Adaptation of The Indonesian Version Functional Assessment of Denture Instrument as a Measuring Tool for Complete Denture Functional Quality

**DOI:** 10.1055/s-0044-1786843

**Published:** 2024-07-19

**Authors:** Arrad Ibrahim Rambey, Nina Ariani, Lindawati Soetanto Kusdhany

**Affiliations:** 1 Prosthodontics Residency Program, Faculty of Dentistry, Universitas Indonesia, Jakarta, Indonesia; 2 Department of Prosthodontics, Faculty of Dentistry, Universitas Indonesia, Jakarta, Indonesia

**Keywords:** complete dentures, edentulous mouth, denture quality, satisfaction, adaptation period of 1–2 weeks

## Abstract

**Objective** Making good quality dentures is necessary to avoid patients' discomfort when adapting to new dentures. Several studies regarding the assessment of the quality of dentures have been carried out in many other countries, such as using the Functional Assessment of Dentures (FAD) by Anastassiadou. However, studies have yet to be conducted in Indonesia. This study aims to obtain a valid and reliable instrument to measure the quality of complete dentures (CDs). This study also aims to find out whether the functional quality of a CD measured with the Indonesian version of the FAD Instrument (PFGT) can affect patient satisfaction in receiving CD treatment (measured with Indonesian version of Patient's Denture Assessment [PDA-Id]) and to find contributing factors to the functional quality of a CD.

**Materials and Methods** The study was conducted in two stages. The first stage was a qualitative study using cross-cultural adaptation methods and focus group discussions with experts. The second stage was a quantitative test with a total of 40 subjects for statistical analysis in the form of a kappa test, test–retest, Kuder–Richardson (KR) 20, and a correlation test between PDA-Id and PFG. Multivariate analysis was done to analyze contributing factors to the functional quality of CD.

**Results** The final instrument was obtained from the qualitative test, which was tested for content and face validation. The interrater kappa test result of 0.828 shows an almost perfect agreement. The results of the intrarater test–retest (0.564; *p* > 0.05; intraclass correlation coefficient 0.889) showed excellent instrument stability. The results of the internal consistency test with Kuder–Richardson 20 (1.08; KR > 1) showed good internal consistency. The correlation test results between PFGT and PDA-Id (0.044; *p* < 0.05) showed a positive correlation. Multivariate analysis showed a relationship between the quality of the CD, the length of time the CD was used, and the patient's satisfaction with CD treatment.

**Conclusion** The PFGT instrument is considered a valid and reliable tool to measure the functional quality of a denture that can distinguish between good and bad quality.

## Introduction

Edentulism can affect the health of the patient's oral cavity and the patient's health in general. Oral conditions affected by edentulism range from resorption of residual ridges to impaired masticatory function, unhealthy diet, social disability, and poor oral health quality of life. 1 Edentulism requires treatment in the form of making dentures such as fixed dentures, implant-supported dentures, removable partial dentures, or complete dentures (CDs), which can improve masticatory abilities and oral health and have an impact on improving the patient's quality of life. The use of dentures may help the problems faced by patients who experience edentulism. However, it is not uncommon for patients to complain of discomfort about the dentures they are using, which are generally related to the retention and stabilization of the dentures. 2 To avoid the discomfort patients feel when they have to adapt to their new dentures, it is necessary to evaluate the quality of a denture first. There is a method discovered by Basker and colleagues and developed by Corrigan et al and Anastassiadou et al in a different study called Functional Assessment of Dentures (FAD). 3 4 5 6

Treatment with conventional CD has been proven successful in providing satisfaction to the majority of patients. However, some patients remain dissatisfied with their prostheses, regardless of the quality of the denture itself. 7 Komagamine et al stated that the success of denture treatment is not dependent on examination by a dentist, but is based on the assessment of the patient who uses the denture itself. 8 In that study, the level of patient satisfaction with denture treatment was measured by a Patient's Denture Assessment (PDA) 8 questionnaire ( Table 1 ) using a Visual Analogue Scale (VAS), which was later developed by Rezeki et al in 2017 to become the Indonesian version of the PDA (PDA-Id). 9

**Table 1:** Patient's Denture Assessment (PDA)

Subscale	Questionnaire items
Function	Q1. How much pain do you feel?
	Q2. How easy it is for you to swallow food boluses and water?
	Q3 How well do you enjoy meals?
	Q4. How worn out does your jaw feel?
Aesthetics and speech	Q5. How worried are you about other people watching?
	Q6. How easy is it for you to speak?
	Q7. How worried are you about your mouth?
	Q8. How often do your dentures click when chewing?
Lower denture	Q9. How often does food debris get stuck under your lower denture?
	Q10. How is your lower denture retained on the ridge?
	Q11. How does your lower denture fit?
	Q12. How uncomfortable is your lower denture?
Expectation	Q13. How satisfactory will the new dentures be?
	Q14. How problematic will the new dentures be?
	Q15. How well will the new dentures fit?
Upper denture	Q16. How often does food debris get stuck under your upper denture?
	Q17. How does your upper denture fit?
	Q18. How often does your upper denture fall down?
Importance	Q19. How much do you consider your dentures as part of your body?
	Q20. How important are your dentures to you?
	Q21. How much can you care for your dentures without any difficulty?
	Q22. How at ease do you feel when wearing your dentures?

Patient satisfaction with a conventional CD treatment is influenced by several factors, apart from the quality of the denture itself. Kapur believes that the quality of the supporting tissue of a denture can also affect the outcome of the treatment performed. 6 10 Assessment of the quality of a CD is not only limited to the condition of the CD but can also be influenced by the condition of the patient's intraoral tissue, such as the shape of the residual ridge, tissue resistance, and the depth of the vestibule can affect the adaptation of the denture base, that it can affect the assessment of the quality of dentures. 10

The main objective of this study was to obtain a valid and reliable Indonesian version of the FAD instrument called Indonesian Version FAD (PFGT) so that it can be used as a valid and reliable measurement tool. This study also aims to find out whether the functional quality of a CD (measured with PFGT) can affect patient satisfaction (measured with PDA-Id) in receiving CD treatment and also to analyze contributing factors to the functional quality of a CD (sociodemographics, length of time the CD was used, and the quality of the denture-supporting tissue).

## Methods

The research consists of two parts, namely, qualitative research and quantitative research. Qualitative research was conducted in the form of cross-cultural adaptation methods with translation, backward translation, and expert discussion. 11 Quantitative research was conducted to test the validity and reliability of the instruments developed with a cross-sectional design and to test the correlation between the instruments developed against the PDA-Id questionnaire. This research was approved by the Ethical Committee from the Faculty of Dentistry, Universitas Indonesia, Jakarta, Indonesia.

Qualitative research begins with cross-cultural adaptation of the FAD instrument ( Table 2 ), by forming two teams of translators, namely, the first team to translate the FAD into Indonesian and the second team to translate the results of the FAD translation back into English. The first team consisted of two clinicians who spoke fluent Indonesian and understood English, producing the T-1 and T-2 instruments. After obtaining the translation results from the two translators, a synthesis was carried out to obtain the T-12 instrument. Then, a second team, consisting of two clinicians fluent in English and understanding Indonesian, retranslated the T-12 instrument, which was then compared with the original FAD instrument to assess whether there was a change in the meaning of the translation results, and a prefinal instrument was obtained.

**Table 2:** Functional Assessment of Denture by Anastassiadou

No.	Parameter	Score (1st)/(2nd)
1	Freeway space (FWS) (Resting vertical dimension [RVD] measured with lower denture *in situ* )	• Adequate = 1 • Wrong = 0
2i	Occlusion The patient is asked to relax and close gently on back teeth several times from a slightly open (20 mm) position.	• Balanced = 1 • Slide = 0
2ii	Articulation Lower jaw moved side to side with teeth lightly together. Observe relationship of denture bases to underlying tissues.	• Minimal displacement = 1 • Excessive displacement = 0
3i	Upper retention (resistance to vertical pull) The mouth is opened 20 mm. Note if denture drops. *With the mouth still open, the denture is grasped by the thumb and index finger on the premolars and a downward force applied. (Dry teeth if necessary.) *Repeat if tongue is in a guarded position	• Adequate resistance = 1 • No Resistance = 0
3ii	Upper retention/Tongue control, incision test A cotton wool roll is inserted between the front teeth and the patient is instructed to close gently onto the roll and bite as if it were a piece of food. The position of the tongue is noted. Judgment made on third attempt	• Upper denture is stabilized by tongue = 1 • Tongue remains in floor of the mouth = 0
4i	Upper stability (lateral displacement) Denture is grasped by the thumb and index finger in the premolar region and a rotational force applied	Lateral displacement • No = 1 • Yes = 0
4ii	Upper stability (pronounced rocking) Light force is applied on the right and left sides in the first molar simultaneously. Attempt to tip in anteroposterior direction with thumb and index finger placed posteriorly and anteriorly simultaneously	Pronounced movement • No = 1 • Yes = 0
5i	Lower stability (displacement) The mouth is opened 20 mm with tongue in relaxed position. Seating of denture checked with fingers	• Lower denture stays in place = 1 • Lower denture is noticeably displaced = 0
5ii	Lower stability (pronounced movement) The patient is instructed to move the tongue so the tip gently resist at the angels of the mouth with the mouth opened 20 mm. Check seating of denture with fingers. Judgment made on third attempt	Pronounced movement • No = 1 • Yes = 0
5iii	Lower stability (anteroposterior movement) Upper denture removed. The lower denture is held against the ridge by a finger and thumb on the incisors and attempt made to move it with tongue in relaxed position	Anteroposterior movement • No = 1 • Yes = 0

After obtaining the prefinal instrument, a pretest was conducted using the purposive sampling method on dentists with experience in prosthodontics (prosthodontic residents or prosthodontists). The number of subjects is 10 dentists 12 who have experience in the field of prosthodontics (prosthodontic resident or prosthodontist), using the unstructured interview method to obtain the final instrument, which was called the PFGT. The final instrument was subjected to advanced validation to evaluate whether the instructions given to the instrument were reasonable, unambiguous, and straightforward.

Quantitative research was conducted using consecutive sampling method in patients who have received conventional CD treatment. Inclusion criteria in this study are patients receiving CD treatment at the Dental Hospital, Faculty of Dentistry, Universitas Indonesia, aged 45 years and older, who agreed to fill out informed consent, and can communicate. Exclusion criteria were CDs made by dental technicians, patients with motoric disability, neurologic conditions, or dementia, and patients who did not want to participate in the study. Based on the sample size calculation using G-Power, the required sample was 40 subjects. Sociodemographics data, the time the CD was used, and the quality of the denture-supporting tissue were obtained from the subjects.

Respondents willing to become research subjects signed an informed consent form; then, data was collected. Subjects filled out the PDA-Id questionnaire and then filled in the PFGT instrument by two examiners who were experienced dentists in the field of prosthodontics and had been calibrated beforehand to be able to fill out questionnaires and instruments. The PFGT was then tested for test–retest reliability with a subsample of 10 subjects who would be reexamined in the same way within 7 to 10 days after the first data collection, and the two examiners collected the data.

Samples are divided into six different groups based on age (between 45 and 59 years old), sex (male or female), length of time the CD was used (0–6 months, or more than 6 months), quality of denture-bearing area (bad, moderate, or good), patient satisfaction (satisfied or not), and the quality of the CD (good or bad).

The statistical package SPSS was used to analyze the data in this research. Cohen's kappa test performed the interexaminer agreement for all data, while the intraexaminer agreement was performed by test–retest reliability analysis. The instrument was then tested for internal consistency by performing a Kuder–Richardson (KR) 20 test and construct validity by performing a Spearman's correlation test compared to PDA-Id.

## Result

The validation team decided to use a credible private language translation agency to translate the original FAD instruments used by the clinicians.

Data was collected by two examiners who were experienced dentists in prosthodontics from 40 subjects, with 21 male and 19 female patients, 20 first-time denture users and 20 previous denture users. The interrater reliability (kappa) results obtained from each item of the PFGT instrument generally had a very good value, while the overall kappa value was 0.828 (almost perfect agreement).

An instrument stability test was carried out with a subsample of 10 subjects, which would be reexamined in the same way within 7 to 10 days after the first data collection. In the test–retest results, a *p* -value of 0.564 ( *p* > 0.05) was obtained, meaning there was no difference in the total score of the first and second observations. The intraclass correlation coefficient (ICC) score obtained from the two examiners was 0.894, based on Oremus et al. If the ICC is above 0.750, it can be said that the stability of the instrument is excellent. 13

Then, an internal consistency test was carried out with the Kuder–Richardson 20 test, and a KR value of 1.08 was obtained, where the instrument's internal consistency was considered very good. The distribution of the data was not normal; the Spearman's correlation test was carried out to validate the convergent validation of the PFGT instrument with the PDA-Id comparator. Based on the results of the Spearman's test, there is a statistically significant positive correlation between the PFGT instrument and the PDA-Id questionnaire ( *p* < 0.05); the correlation value (0.32) is considered fair based on Chan. 14

The area under the receiver operator characteristic curve (AUROC) value of the PFGT instrument gets a poor interpretation, which means it is relatively weak when correlated with PDA-Id but can still be declared valid because the AUROC value is > 0.5 (0.659). A cutoff value with a sensitivity of 70% and a specificity of 60% is still acceptable, with a value of 8.5. Furthermore, an analysis was performed on factors related to denture quality, such as age, gender, length of time the CD was used, quality of the CD support network, and patient satisfaction with CD treatment as measured by PDA-Id.

Based on the chi-square bivariate analysis ( Table 3 ), significant factors related to denture quality were the length of time the CD was used and patient satisfaction with CD treatment as measured by PDI-Id ( *p* < 0.05). CD usage duration of 0 to 6 months is more likely to produce good quality CD (odds ratio [OR] 0.21, 95% confidence interval [CI] 0.05–1.01, *p* = 0.042). Patient satisfaction with CD treatment as measured by PDA-Id had a 12.31 times probability of receiving good quality CD (95% CI OR 1.39–109.10; *p* = 0.008). Furthermore, variables with *p* -values < 0.25 are entered as multivariate candidates.

**Table 3:** Bivariate analysis of the relationship between denture quality scores using the PFGT instrument, and factors such as age, gender, the length of time the CD was used, and quality of denture supporting tissues

Variable	Code	Category	Bad (score < 8.5) *N* = 17		Good (score ≥ 8.5) *N* = 23		Total	*p* -Value	OR	95% CI OR	
			*n*	%	*n*	%					
Age	0	45–59 years old	6	40.0	9	60.0	15	0.804	1.00		
	1	> 60 years old	11	44.0	14	56.0	25		0.85	0.23	3.11
Gender	0	Male	7	33.3	14	66.7	21	0.218	1.00		
	1	Female	10	52.6	9	47.4	19		0.45	0.13	1.62
The length of time the CD was used	0	0–6 mo	10	33.3	20	66.7	30	0.042 a	1.00		
	1	> 6 mo	7	70.0	3	30.0	10		0.21	0.05	1.01
Quality of denture-supporting tissue	0	Bad (score < 14)	8	53.3	7	46.7	15	0.515	1.00		
	1	Moderate (score 14–17)	8	34.8	15	65.2	23		2.14	.58	8.09
	2	Good (score > 17)	1	50.0	1	50.0	2		1.14	.06	21.87
Patient satisfaction with CD treatment (PDA-Id)	0	Dissatisfied (< 2040)	16	55.2	13	44.8	29	0.008 a	1.00		
	1	Satisfied (≥ 2040)	1	9.1	10	90.9	11		12.31	1.39	109.10

Abbreviations: CD, complete denture; CI, confidence interval; OR, odds ratio; PDA-Id, Indonesian version of Patient's Denture Assessment; PFGT, Indonesian Version Functional Assessment of Dentures.

a *p* < 0.05.

The results of multivariate analysis with logistic regression ( Table 4 ) showed that what affected the quality of the denture was the length of time CD was used and the patient's satisfaction with CD treatment. The final result is a model that only consists of two variables: the length of time the CD was used and patients who are satisfied with CD treatment. The magnitude of the influence of these variables is that the duration of using a CD for “0 to 6 months” has a greater probability of getting a CD with good quality (OR 0.215, 95% CI 0.041–1.127, *p* 0.069). Patient satisfaction with CD treatment who was “satisfied” had a risk of 4.487 times (95% CI OR 1.078–18.668; *p* = 0.039) to get good-quality dentures. From the results of the multivariate analysis, it can be said that the most significant influence on denture quality measured using the PFGT instrument is “patient satisfaction,” as measured using the PDA-Id with an OR of 4.487.

**Table 4:** Multivariate analysis (final model) of the relationship between denture quality using the PFGT instrument and sociodemographic factors, the length of time the CD was used, and the quality of denture supporting tissue

Variable	Category	Coefficient	SE	*p* -Value	OR	95% CI OR	
						Lower	Upper
The length of time the CD was used	> 6 mo vs. 0–6 mo	–1.537	0.845	0.069 b	0.215	0.041	1.127
Patient satisfaction with CD treatment (PDA-Id)	Satisfied (≥ 2040) vs. Dissatisfied (< 2040)	1.501	0.727	0.039 a	4.487	1.078	18.668
Constant		–0.14	0.507	0.978	0.986		

Abbreviations: CD, complete denture; CI, confidence interval; OR, odds ratio; PDA-Id, Indonesian version of Patient's Denture Assessment; PFGT, Indonesian Version Functional Assessment of Dentures; SE, standard error.

a *p* < 0.05.

b *p* < 0.1.

## Discussion

As Beaton et al explained in his guide to cross-cultural adaptation, an instrument used in another country with a different language should require cross-cultural adaptation to ensure consistent content validity between the source and target instrument being developed. 11 Several questions in this instrument cannot be translated directly from English to Indonesian due to cultural differences, such as the use of 3 to 7 mm freeway space (FWS) used by previous researchers, 3 4 6 7 where the common FWS used in the researcher region is 2 to 4 mm. 15

The Spearman's correlation test was carried out on the PFGT instrument on the PDA-Id questionnaire instrument with a *p* -value of 0.044 ( *p* < 0.05), indicating a correlation between the PFGT instrument and the PDA-Id questionnaire. However, the Spearman's correlation coefficient obtained was 0.320 (0.3–0.4 = fair), which indicates a positive correlation that is not strong from this assessment. From the results of the correlation test, based on the interpretation of Chan, 14 it can be concluded that there is a weak correlation between patient satisfaction with denture treatment and denture quality. This result is supported by several other studies stating that many factors influence patient satisfaction with dentures, such as patient expectations and psychological factors. 3 16 17 18 According to Carlsson, patients with too high expectations are at risk of experiencing dissatisfaction with the CD treatment they receive, so the dentist's job here is to adjust the patient's expectations of CD treatment and explain all the limitations of CD compared to natural teeth. 17 Guckes, in his research, showed that patient satisfaction with the CD received correlated with the patient's opinion of the denture, where this can be easily handled if a counseling session is held with the dentist to improve the patient's opinion of the CD treatment, as well as lowering patient expectations. 19 Another factor influencing patient satisfaction with dentures is psychological factors; 16% of patients still complain about their CD even though the quality of the CD is considered good enough. 18 This is closely related to neuroticism, a condition in which the patient is always disposed to have negative thoughts about everything that happens in their life. 20

Although it has been widely reported that patients may be dissatisfied with the CD care they receive, even though the quality of the CD is considered good, the opposite can also happen, where the quality of the CD is considered lower than the standards. However, patients are still satisfied with the CD they received. This was revealed by Carlsson, that “a good relationship between doctor and patient is more important than a suitable denture manufacturing procedure, in getting patient satisfaction.” 16 This shows that good communication between doctors and patients is one of the primary keys in achieving patient satisfaction with the CD treatment received.

Some literature states that many factors can influence patient satisfaction with denture care. However, in this study, we wanted to know what factors could have a relationship with the quality of a denture. A chi-square bivariate test was performed with factors such as age, gender, the length of time the CD was used, quality of denture-supporting tissue, and patient satisfaction with dentures. The test results found that the length of time the CD was used was related to the denture's functional quality. This is in line with Ribeiro et al's research, where the quality of a denture will continue to decline over time. 21 In Leles et al's study, it was found that denture quality improved after 3 months of use but decreased after 6 months. This is because the patient underwent a process of adaptation to dentures during the first 3 months, but the supporting tissue of the dentures underwent changes after using CD for 6 months. 6 Further research is needed regarding the relationship between the quality of dentures and the age of dentures due to the limitations of this study, which are only cross-sectional, so there is no assessment of intraindividual changes over time, whether there is an increase or decrease in quality according to previous research.

As previously discussed, patient satisfaction with dentures has a relationship with denture quality, which indicates a relationship between patient satisfaction with dentures and denture quality, although the Spearman's correlation test proved to have a weak relationship. From the results of multivariate analysis, it was found that patient satisfaction with CD treatment had an OR of 4.487, which means that patients who are satisfied with their CD are 4.487 times more likely to get a good-quality CD. This is in line with the results of the Celebić et al study in 2003, which stated that denture quality has a strong correlation with patient satisfaction, besides other factors such as education level, economic status, and quality of life. 22

In future studies, it is necessary to conduct a prospective study to assess the correlation between denture quality and wear over time by comparing new and old dentures in the same individual. In other studies by Cerutti-Kopplin et al, it is also necessary to relate to other factors like retention, comfort, esthetic, phonetics, and different adaptation periods. 7 23 In addition, in future studies, illustrations or videos should be provided to give an overview of the operators' examinations.

## Conclusion

Quantitative research has succeeded in testing the validity and reliability of the instruments that have been developed, as well as proving a positive correlation between CD quality and patient satisfaction with CD treatment. In addition, this study also showed a relationship between the quality of the CD and the length of time the CD was used. However, this study found that there was no relationship between sociodemographics and the supporting tissue quality, on the quality of CD.

This instrument will later become a measuring tool widely used to obtain further research data and can help to be considered for decision-making whether a patient needs a new CD or just some repairs to the old one.
